# Wine glass size and wine sales: four replication studies in one restaurant and two bars

**DOI:** 10.1186/s13104-019-4477-8

**Published:** 2019-07-17

**Authors:** Natasha Clarke, Rachel Pechey, Mark Pilling, Gareth J. Hollands, Eleni Mantzari, Theresa M. Marteau

**Affiliations:** Behaviour and Health Research Unit, University of Cambridge, Cambridge, UK

**Keywords:** Wine, Alcohol, Sales, Purchasing, Glass size, Replication, Multiple treatment reversal design, Bar, Restaurant

## Abstract

**Objective:**

Previous research suggests that wine glass size affects sales of wine in bars, with more wine purchased when served in larger glasses. The current four studies, conducted in one restaurant (Studies 1 and 2) and two bars (Studies 3 and 4) in Cambridge, England, aim to establish the reproducibility of this effect of glass size on sales. A multiple treatment reversal design was used, involving wine being served in sequential fortnightly periods in different sized glasses of the same design (290 ml, 350 ml, and 450 ml). The primary outcome was daily wine volume (ml) sold.

**Results:**

Restaurant: Daily wine volume sold was 13% (95% CI 2%, 24%) higher when served with 350 ml vs. 290 ml glasses in Study 1. A similar direction of effect was seen in Study 2 (6%; 95% CI − 1%, 15%). Bars: Daily wine volume sold was 21% (95% CI 9%, 35%) higher when served with 450 ml vs. 350 ml glasses in Study 3. This effect was not observed in Study 4 (− 7%, 95% CI − 16%, 3%). Meaningful differences were not demonstrated with any other glass comparison. These results partially replicate previous studies showing that larger glasses increase wine sales. Considerable uncertainty remains about the magnitude of any effect and the contexts in which it might occur.

*Trial registration* Study 1: ISRCTN17958895 (21/07/2017), Study 2: ISRCTN17097810 (29/03/2018), Study 3 and 4: ISRCTN39401124 (10/05/2018)

**Electronic supplementary material:**

The online version of this article (10.1186/s13104-019-4477-8) contains supplementary material, which is available to authorized users.

## Introduction

Alcohol consumption is a leading risk factor for global disease burden worldwide [1] and is the fifth leading factor in the UK [2]. One potential intervention to reduce alcohol consumption is to change the size of glassware [3]. Larger glasses may increase consumption by two means. First, by influencing pouring behaviour, with larger glasses resulting in more alcohol being poured into them [4, 5]. Second, by influencing perceptions of volume, with the same volume of alcohol being perceived as less when poured into larger compared with smaller glasses [6]. As people tend to consume in units—one glass of wine, one slice of cake—known as “*the unit bias heuristic*” [7], if a serving of wine is perceived as *less than a glass* it could lead some people to drink another glass.

This replication paper extends previous studies that indicate an effect of glass size on sales, a proxy measure of consumption, in restaurant and bar settings in Cambridge, England [8, 9]. The first of these studies, carried out in a single establishment that had separate bar and restaurant areas, found that serving wine in larger (370 ml capacity) compared with medium-sized (300 ml) glasses, keeping serving size constant, increased wine sales by 9.4%. This difference in sales was 14.3% in the bar area, compared to a non-significant difference of 8.2% in the restaurant. Results were inconclusive when comparing sales using smaller glasses (250 ml) with medium-sized glasses (300 ml) [8].

The second study was carried out in two bars and used glass sizes with capacities of 300 ml, 370 ml, 510 ml (Bar 1) and 300 ml and 510 ml (Bar 2). In Bar 1, daily wine volume purchased was 11% higher when sold in 510 ml compared to 370 ml glasses. Findings were inconclusive for the other glass comparisons [9]. These results provided a partial replication of the initial study, showing that introducing larger glasses increased sales. However, the pattern of results was mixed, which could reflect a moderating influence, such as the serving size selected, characteristics of the establishment, such as differences in sales by bottle vs. glass, or random fluctuations rather than true effects. The current paper aims to establish the reproducibility of an effect of glass size on sales in four studies, conducted in one restaurant and two bars in England.

## Main text

### Methods

#### Study design

Wine glasses of different sizes i.e. bowl capacities, were changed over fortnightly periods in each establishment in a multiple treatment reversal design using 290 ml, 350 ml and 450 ml glasses (see Table 1). The primary outcome was the daily volume of wine (ml) sold. Reference groups were 290 ml for the restaurant and 350 ml for the bars.

**Table 1:** Glass capacity (ml) by fortnightly period for each study

Fortnight	Study 1 (Restaurant A)	Study 2 (Restaurant A)	Study 3 (Bar A)	Study 4 (Bar B)
1	290	290	350	350
2	350	350	290	290
3	290^a^	290	350	350
4	450	450	450	450^b^
5	290	290	350	350
6	350	350	290	290
7	290	290	350^b^	350
8	450	450^b^	450	450
9	290	290	350	350
10		350		
11		290		
12		450		
13		290		

^a^Following Fortnight 3, there was an excluded 2-week period for Study 1, when 230 ml glasses (rather than the 450 ml) were introduced to better match the glasses in the initial study on wine glass size (7), but these were withdrawn following customer complaints

^b^Due to fidelity check violations (i.e. glasses not changed over on time), these periods lasted 3 weeks with establishments continuing to use this glass size for one extra week, although these additional weeks were not included in analyses

#### Intervention

The glasses used were of the same design (Royal Leerdam Bouquet), with capacities of 290 ml, 350 ml and 450 ml. These were of a slightly different design and larger capacities than glasses used in the initial study [8] (Royal Leerdam Fortius 250 ml, 300 ml and 370 ml) due to the range being discontinued. The sizes compared in the different settings were constrained by the serving sizes offered in the participating establishments.

In keeping with UK law [10], serving sizes offered were not altered. All establishments offered wine by the glass in 125 ml and 175 ml serving sizes and 75 cl bottles. The restaurant also offered wine in 50 cl and 100 cl carafes. The 75 cl bottles and 50/100 cl carafes were free-poured into glasses by bar staff or customers. The bars—but not the restaurant—also offered wine in 250 ml serving sizes.

Changing the size of wine glasses is categorised as a Size × Product intervention within the TIPPME (Typology of Interventions in Proximal Physical Micro-Environments) [11].

#### Setting

The study was conducted in one independent restaurant (Study 1 and 2) and two bars from the same pub group (Study 3 and 4) in Cambridge, England. One size of wine glass was used at any one time for all wine sold regardless of serving size, with the exception of sparkling wines, sales of which were excluded from the current studies. See Table 2 for establishment characteristics.

**Table 2:** Characteristics of participating establishments

	Previous studies			Current studies			
	Bar and restaurant	Bar 1^a^	Bar 2	Restaurant A		Bar A	Bar B
	Pechey et al. [8]	Pechey et al. [9]		Study 1	Study 2	Study 3	Study 4
Standard glass size (ml)	300	350	350	310^b^	350^c^	350	350
Intervention glass sizes (ml)	250, 300, 370	300, 370, 510	300, 510	290, 350, 450	290, 350, 450	290, 350, 450	290, 350, 450
Price of 175 ml of wine (£)	5.00	4.10	5.40	5.90	5.90	5.21	4.34
Serving sizes offered (ml)							
Fixed	125, 175	125, 175, 250	125, 175, 250	125, 175	125, 175	125, 175, 250	125, 175, 250
Free-poured	500, 750, 1000	750	750	500, 750, 1000	500, 750, 1000	750	750
Sales by-the-glass (%)	Bar: 93 Restaurant: 63	88	88	66	67	90	91
Study period	March–July 2015	March–July 2016	March–July 2016	July–November 2017	April–October 2018	May–September 2018	May–September 2018

^a^Bar 1 in the previous studies is the same establishment as Bar A in the current studies

^b^In Study 1, wine glasses in current use in establishments were replaced in order to compare sales of wine when using 350 ml glasses to sales when using 450 ml and 290 ml glasses

^c^Following Study 1, this establishment updated their standard glass size from 310 ml to 350 ml Royal Leerdam Bouquet glasses

#### Procedure

Glasses were changed by bar or restaurant staff in each of the participating establishments on Monday mornings each fortnight throughout the study period. Email reminders were sent by a researcher at 8 a.m. on the morning of a glass changeover. The manager of each establishment was asked to confirm that the glass change had occurred prior to opening that day. Fidelity to protocol was checked by a researcher visiting the restaurant or bar at the start of each fortnightly period. The check involved a person from the research group acting as a customer and first, checking that the glasses in the bar area were all of the same design and second, buying a small glass of wine (125 ml). The checker then measured the height of the wine glass to ensure it matched the scheduled glass size. In the case of violations, the lead researcher contacted the manager to report the violation. Glass changeover then took place as soon as possible, and the actual date of changeover recorded. If the establishment’s manager could not be reached to confirm when a changeover occurred following a protocol violation, that week’s data was not included in analyses, and the establishment continued using the affected glass size for one extra week assuming the next fidelity checks were passed. Sales data were obtained from the till records of each venue.

#### Data analysis

Protocol violations were recorded on eight out of 40 fidelity checks - one occasion in Study 2, two in Study 3, and five in Study 4. For three of these (one in each study), data collection was delayed by a week due to being unable to confirm changeover. In keeping with our plan of analysis, data for these three weeks were not used in the analyses.

Regression analyses predicted daily wine sales volume (ml) from glass size, modelled using dummy variables. Natural log was used as a variance stabilising transformation.

See Additional file 1 for further details on data analysis.

### Results

**Fig. 1:**
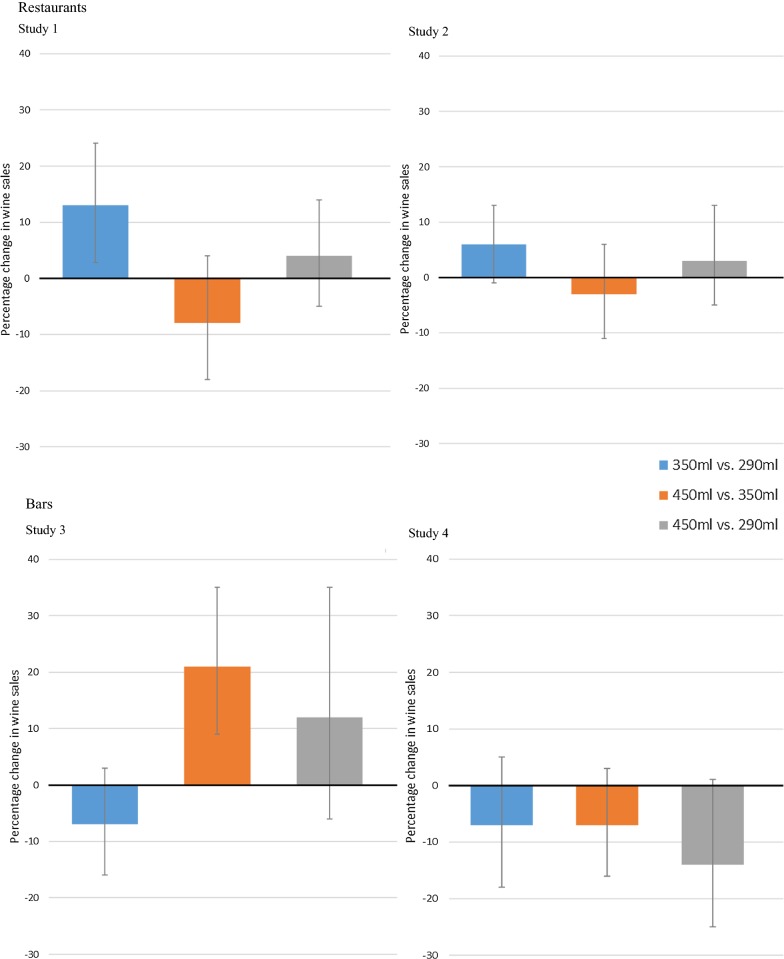
Change (%) in wine sales in A. Restaurants and B. Bars, by wine glass size comparison (error bars with 95% CI)

Figure 1 shows the results of the main regression analyses for each comparison in each establishment (separated by restaurant and bars), controlling for various covariates. See Additional file 2 for unadjusted mean sales volume for each establishment under the different glass size conditions.

#### Restaurant

In the restaurant for Study 1, daily wine sales were 12.6% (95% confidence interval (CI) 2.4% to 23.7%) higher when 350 ml glasses were used compared to 290 ml glasses. A similar direction of effect was seen in Study 2 but was not statistically significant (6.3% sales increase; 95% CI − 1.4%, 14.7%). Daily wine sales were not significantly different in the restaurant when using 450 ml glasses compared to 350 ml glasses (Study 1: 7.6% decrease, 95% CI − 17.7%, 3.8%; Study 2: 2.7% decrease, 95% CI − 10.6%, 5.9%). Daily wine glass sales were not significantly different when comparing 450 ml glasses to 290 ml glasses (Study 1: 4.1% increase, 95% CI − 5.0%, 14.0%; Study 2: 3.4% increase, 95% CI − 5.1%, 12.8%).

#### Bars

In Bar A (Study 3), daily wine sales were 21.4% (95% CI 8.8%, 35.3%) higher when sold using 450 ml glasses compared to 350 ml glasses. This effect was not observed in Bar B (Study 4), (6.9% sales decrease, 95% CI − 17.7%, 5.4%). Daily wine sales were not significantly different in the bars when comparing 350 ml glasses with 290 ml glasses (Study 3: 7.4% decrease, 95% CI − 21.6%, 9.5%; Study 4: 7.2% decrease, 95% CI − 16.5%, 2.9%). Daily wine glass sales were not significantly different when comparing 450 ml glasses to 290 ml glasses (Study 3: 12.4% increase, 95% CI − 6.3%, 34.9%; Study 4: − 13.6% decrease, 95% CI − 25.4%, 0%).

### Discussion

In the restaurant, sales of wine increased by 13% when served using 350 ml glasses compared to 290 ml in Study 1. This was not replicated in Study 2, and no other comparisons in the restaurant revealed any meaningful difference in sales. In the bars, sales of wine increased by 21% when served using 450 ml glasses compared to 350 ml glasses in Study 3. This was not replicated in Study 4 and no other comparisons in the bars revealed any meaningful differences in sales. Although the expected association was absent in most of the comparisons, these results indicate a partial replication of previous studies showing that larger glasses increase wine sales (7, 8), as the only significant differences were in the expected direction and there was no evidence of decreases in sales with larger glasses. Previous studies have found similar inconsistencies, thus considerable uncertainty remains about the magnitude of the overall effects and the contexts in which they might occur.

There are a number of possible explanations for the inconsistent effects of glass size—across establishments and by glass comparison—observed in these studies. These include random fluctuations and context effects. First, it may be that increases in sales are wholly or partly a result of random fluctuations in purchasing behaviour, rather than representing effects of the intervention i.e. glass size. But if this were the case, some significant decreases in sales could also be expected, which is not evident from the results in the current or indeed previous studies.

Second, it may be that sales patterns are context-dependent and differ with characteristics of venues or, more broadly, between restaurants and bars. More wine was sold by the bottle in the restaurant than in the bars: 33% vs. 10%. The effect of wine glass size may be greater in the context of wine freely poured from a bottle or carafe by staff or customers. In keeping with this, pouring inaccuracy increases with size of wine glass with larger glasses leading to more wine being poured [5]. Further research is required to assess this possible explanation, including examination of pouring inaccuracies with different glass sizes in naturalistic drinking settings.

Third, inconsistent effects may reflect differences between settings in available serving sizes. When purchasing by the glass, customers purchase a fixed measure of wine, thus any influence of glass size may be due to perceptual differences. In the restaurant, fixed measures of 125 ml and 175 ml were available, and the significant increase in sales occurred when comparing 350 ml glasses to 250 ml glasses. In the bars, fixed measures of 125 ml, 175 ml and 250 ml were available, and the significant increase occurred when comparing 450 ml glasses to 350 ml glasses. Similarly, in one of these bars an increase of 10% was found with similar glass sizes (510 ml vs. 370 ml) in a previous study [8]. This might reflect an interaction between wine glass size and serving size, with perceptions of various serving sizes differing by glass size.

#### Implications for research and policy

Considerable uncertainty remains around the exact conditions under which larger wine glasses might increase sales given the current and previous findings. Given this uncertainty, reflecting a large number of different comparisons, a meta-analysis of all previous studies is an important next step in clarifying the effects on sales of altering wine glass size in restaurants and bars.

### Conclusions

These results provide a partial replication of previous studies, generating some evidence in support of larger glasses increasing sales of wine under certain conditions and no evidence of an effect in the opposite direction. Further research is required to clarify the effects of glass size, the conditions under which the effect is largest and the mechanisms by which glass size influences sales.

## Limitations

The strength of the studies presented here is that they add evidence to that generated from three previous field studies published in two articles, in a bar and restaurant setting [8] and two bars [9]. All these studies used objective outcomes assessing the impact of glass size on sales and considering potential covariates. The current research replicates the study design and method in the same bar (Study 3) as one of the previous studies [9] and in two further establishments, with a greater range of glass comparisons.

The results need to be interpreted in the context of several limitations. First, the primary outcome measure was wine sales, not actual consumption. Purchasing behaviour is, however, a valid proxy of consumption [8, 9] and a more practicable objective outcome measure in field settings [12]. Second, due to the nature of the field study, it was not possible to record characteristics of customers or details of their behaviour in the establishments. Third, the studies were conducted in a relatively affluent area of England, requiring more studies in other settings and populations. Finally, due to commercial constraints slightly different glass sizes were used in the current four studies compared to previous studies (250 ml, 300 ml, 370 ml [7]; 300 ml, 370 ml and 510 ml [8]). The lack of exact replications of the intervention characteristics, while unavoidable, makes it more difficult to compare and interpret findings.

## Additional files

**Additional file 1.** “Detailed analysis outline”—further details on data analysis.

**Additional file 2.** “Daily wine sales (litres per day) for each establishment, by glass size [unadjusted mean (SD)]”—table showing unadjusted means of wine sales in each establishment.

## Data Availability

All studies were pre-registered (Study 1: ISRCTN17958895: 10.1186/ISRCTN17958895; Open Science Framework: https://osf.io/k9ucd/. Study 2: ISRCTN17097810: 10.1186/ISRCTN17097810; Open Science Framework: https://osf.io/653te/. Study 3 and 4: ISRCTN39401124: 10.1186/ISRCTN39401124; Open Science Framework: https://osf.io/m5qb9/). Data are commercially sensitive, and were provided by bars on the understanding that these would not be made available beyond the research team.

## Abbreviations

CI: confidence interval
TIPPME: Typology of Interventions in Proximal Physical Micro-Environments
