# Supplementary material for: Wine glass size and wine sales: four replication studies in one restaurant and two bars
Source: BMC Res Notes. 2019 Jul 17;12:426. doi: 10.1186/s13104-019-4477-8 (PMC6637618; doi:10.1186/s13104-019-4477-8)
Supplement: Supplementary file 1 — Additional file 1. “Detailed analysis outline”—further details on data analysis. [file 13104_2019_4477_MOESM1_ESM.docx]

Additional file 1

**Detailed analysis outline**

Analyses included considering the following variables as potential covariates: dummy variables for day of week, daily temperature, holiday periods (two dummies indicating school holidays and bank holidays), World Cup 2018 football match days and England World Cup 2018 football match games (due to spikes in custom on these days). The number of items sold (excluding wine sales, logged) for each day was used as a proxy for the number of customers, to control for sales fluctuations over time. Heteroscedasticity was examined, to establish if both the mean and variance of wine sales volume should be modelled. Where appropriate higher terms were included. Model diagnostics included residual plots, QQ-plots, worm plots, and model comparison (as sensitivity analyses) used Akaike information criterion (AIC) values~~.~~ In previous studies, the variance of day of week and glass size were required to adequately model the variance due to heteroscedasticity. In the four studies reported here, for modelling the mean all studies required (to minimise the AIC) glass size, log of sales excluding wine (proxy for number of customers), day of the week and week (as a random variable), with some studies also requiring temperature, football event variables and bank holiday / school holiday variables. When modelling variance terms the day of week and week (as a random variable) were required for all studies, with glass size and temperature required for two studies (Study 2 and Study 3) and football event variables required for two studies (Study 3 and Study 4).
