# Supplementary material for: Wine glass size and wine sales: four replication studies in one restaurant and two bars
Source: BMC Res Notes. 2019 Jul 17;12:426. doi: 10.1186/s13104-019-4477-8 (PMC6637618; doi:10.1186/s13104-019-4477-8)
Supplement: Supplementary file 2 — Additional file 2. “Daily wine sales (litres per day) for each establishment, by glass size [unadjusted mean (SD)]”—table showing unadjusted means of wine sales in each establishment. [file 13104_2019_4477_MOESM2_ESM.docx]

Additional file 2

**Daily wine sales (litres per day) for each establishment, by glass size [unadjusted mean (standard deviation [SD])]**

| **Glass size** | **Study 1 (Restaurant A)** | **Study 2 (Restaurant A)** | **Study 3**  **(Bar A)** | **Study 4**  **(Bar B)** |
| --- | --- | --- | --- | --- |
| 290ml | 15.37 (6.79) | 13.33 (7.19) | 5.94 (3.87) | 11.27 (6.52) |
| 350ml | 16.14 (8.16) | 14.19 (6.38) | 6.13 (3.43) | 10.15 (6.52) |
| 450ml | 14.50 (7.12) | 12.98 (6.39) | 5.97 (4.11) | 9.95 (5.45) |
